# A modified Delphi approach to determine current treatment advances for the development of a resuscitation program for maternal cardiac arrest

**DOI:** 10.1186/s12873-022-00704-7

**Published:** 2022-08-26

**Authors:** Andrea D. Shields, Jacqueline D. Battistelli, Laurie B. Kavanagh, Brook A. Thomson, Peter E. Nielsen

**Affiliations:** 1 Department of Obstetrics and Gynecology, University of Connecticut, Farmington, CT USA; 2 Department of Obstetrics and Gynecology, San Antonio Uniformed Services Health Education Consortium, San Antonio, TX USA; 3 Department of Obstetrics and Gynecology, University of Texas Health Sciences Center, San Antonio, TX USA

**Keywords:** Cardiopulmonary resuscitation pregnancy modifications, Maternal cardiac arrest, Resuscitation

## Abstract

**Objective:**

Maternal cardiac arrest is a rare and complex process requiring pregnancy-specific responses and techniques. The goals of this study were to (1) identify, evaluate, and determine the most current best practices to treat this patient population and (2) establish a standardized set of guidelines to serve as a foundation for a future educational simulation-based curriculum.

**Study design:**

We used a three-step modified Delphi process to achieve consensus. Twenty-two healthcare experts from across North America agreed to participate in the expert panel. In round 1, 12 pregnancy-specific best practice statements were distributed to the expert panel. Panelists anonymously ranked these using a 7-point Likert scale and provided feedback. Round 2 consisted of a face-to-face consensus meeting where statements that had not already achieved consensus were discussed and then subsequently voted upon by the panelists.

**Results:**

Through two rounds, we achieved consensus on nine evidence-based pregnancy-specific techniques to optimize response to maternal cardiac arrest. Round one resulted in one of the 12 best practice statements achieving consensus. Round two resulted in six of the remaining 12 gaining consensus. Best practice techniques involved use of point-of care ultrasound, resuscitative cesarean delivery, cardiopulmonary resuscitation techniques, and the use of extracorporeal cardiopulmonary resuscitation.

**Conclusion:**

The results of this study provide the foundation to develop an optimal, long-term strategy to treat cardiac arrest in pregnancy. We propose these nine priorities for standard practice, curricula, and guidelines to treat maternal cardiac arrest and hope they serve as a foundation for a future educational curriculum.

## Background

### Maternal cardiac arrest

Maternal cardiac arrest (MCA) is the final common pathway for many critical illnesses and comorbidities, however, anatomic and physiologic changes specific to pregnancy and the presence of a fetus create a very different situation as compared to non-pregnant patients. Therefore, pregnant patients who suffer cardiac arrest require a modified and multidisciplinary approach to maximize their survival and ensure optimal outcomes for the fetus. Rates of maternal mortality in the United States are increasing [1], and too many women are dying from pregnancy-related complications [2]. Research suggests that 60 % of these deaths are preventable [3], affording a meaningful opportunity to address these devastating outcomes.

Collectively, cardiovascular conditions, cardiomyopathy, and stroke accounted for more than 30% of pregnancy-related deaths from 2011 to 2016 [4]. Although cardiac arrest during delivery is rare [5], the incidence of this clinically challenging scenario may be increasing [4, 6, 7].

In 2015 the American Heart Association (AHA) published its first scientific statement on MCA [3]. However, despite the AHA’s rigorous scientific processes and dissemination, practitioners have not widely implemented these care modifications. In fact, several studies have shown that multidisciplinary teams often completely miss or incorrectly perform multiple key steps to successful MCA resuscitation [8, 9]. The 2015 AHA guidelines did not address extracorporeal cardiopulmonary resuscitation (ECPR) or point of care ultrasound (POC-US) within the setting of MCA. It also refers to the potentially life-saving measure of MCA resuscitation as “perimortem cesarean delivery,” which was originally described as a procedure to perform to save the fetus in the dying mother [10].

Recognizing this practical deficit, the core investigators (A.S, J.B, B.T, P.N.) identified critical gaps in treatment since the publication of the AHA statement, developed a consensus on treatment priorities, and created a foundation for a future simulation-based curriculum to train providers to treat maternal cardiac arrest successfully. The AHA have subsequently updated their guidelines in 2020, and ILCOR updated their guidelines in 2021. While the AHA guidelines do specifically address MCA resuscitation, ILCOR did not. Table 1 highlights several key similarities and differences between the 2020 AHA and 2022 OBLS guidelines.

**Table 1:** Comparison of key similarities and differences of AHA and OBLS maternal cardiac arrest resuscitation guidelines

Category	AHA 2020 Guideline	OBLS 2022 Guideline
Airway management	Because pregnant patients are more prone to hypoxia, oxygenation and airway management should be prioritized during resuscitation from cardiac arrest in pregnancy	Unchanged
Chest compressions	Priorities for the pregnant woman in cardiac arrest should include provision of high-quality CPR and relief of aortocaval compression through left lateral uterine displacement	High quality chest compressions may require hand positioning to rotate toward the patient’s shoulder (while still applying force to the sternum) to accommodate the larger/pendulous breasts of a pregnant patient
Fetal monitoring during CPR	Because of potential interference with maternal resuscitation, fetal monitoring should not be undertaken during cardiac arrest in pregnancy	Fetal monitors should be removed during resuscitation
Cesarean Delivery during CPR (Terminology, Timing, Personnel)	Perimortem cesarean delivery	The term “resuscitative cesarean delivery” should be used instead of “perimortem cesarean delivery” to more correctly describe the purpose/indication and increase the sense of urgency for performing this procedure
	At 4 min, a PMCD should be strongly considered for every mother in whom ROSC has not been achieved after ≈4 minutes of resuscitative efforts with no ROSC	Concur, with the addition that it is reasonable to consider RCD immediately in a term patient in maternal cardiac arrest
	If maternal viability is not possible (through either fatal injury or prolonged pulselessness), the procedure should be started immediately; the team does not have to wait to begin the PMCD	Preform resuscitative caesarean delivery immediately in a pregnant patient with a fundus height at or above the umbilicus with a non-shockable rhythm
	Decisions on the optimal timing of a PMCD for both the infant and mother are complex and require consideration of factors such as the cause of the arrest, maternal pathology and cardiac function, fetal gestational age, and resources (ie, may be delayed until qualified staff is available to perform this procedure). Shorter arrest-to-delivery time is associated with better outcome (Class I; Level of Evidence B).	Unchanged
	Continuous manual LUD should be performed throughout the PMCD until the fetus is delivered (*Class IIa; Level of Evidence C*). Care should be taken to avoid injury to the rescuer performing the manual LUD during PMCD	Unchanged
	If the uterus is difficult to assess (e.g., in the morbidly obese), then determining the size of the uterus may prove difficult. In this situation, PMCD should be considered at the discretion of the obstetrician by using his or her best assessment of the uterus. In these patients, bedside ultrasound may help guide decision making	See POC-US comments
POC-US	We suggest against the use of point-of-care ultrasound for prognostication during CPR (Class 3: No benefit, LOE C-LD). This recommendation does not preclude the use of ultrasound to identify potentially reversible causes of cardiac arrest or detect ROSC	Where available, POC-US should be used in the management of maternal cardiac arrest for identification of an intrauterine pregnancy and quick determination of gestational age to guide decision making on PMCD 1a. Note: POC-US should not interfere with CPR, thus should only be performed during brief pauses in CPR
		POC-US should be considered for use during maternal cardiac arrest in emergency protocols for identification of potentially reversible causes of cardiac arrest, identification of cardiac contractility activity without palpable pulse for clinical reclassification of pulseless electrical activity, and identification of the absence of cardiac contractility where further attempts at resuscitation may be unsuccessful 2a. Note: POC-US should not interfere with CPR, thus should only be performed during brief pauses in CPR
ECPR	Not discussed	The use of ECPR may be considered for management of maternal cardiac arrest when there is no ROSC after resuscitative hysterotomy (if beyond 20 weeks) and the patient is in an ECMO center with the capacity to care for critically ill pregnant patients
		The use of ECPR should be considered for organ procurement in pregnant patients post-arrest with circulatory determination of death
		All pregnant women who receive ECPR in the setting of maternal cardiac arrest, regardless of outcome, should be reported in the Extracorporeal Life Support Organization (ELSO) registry (http://www.elso.org/Registry.aspx)
Targeted temperature management	We recommend targeted temperature management for pregnant women who remain comatose after resuscitation from cardiac arrest	Unchanged
	During targeted temperature management of the pregnant patient, it is recommended that the fetus be continuously monitored for bradycardia as a potential complication, and obstetric and neonatal consultation should be sought	Unchanged
Preparedness	Care teams that may be called upon to manage these situations should develop and practice standard institutional responses to allow for smooth delivery of resuscitative care	Providers staffing emergency departments should be trained in resuscitative cesarean delivery
	Protocols for management of OHCA in pregnancy should be developed to facilitate timely transport to a center with capacity to immediately perform PMCD while providing ongoing resuscitation	A minimum of 3 providers should be utilized during OH MCA resuscitation. It is reasonable to consider the use of automatic chest compression devices (ACCD) to assist with resuscitation with limited resources

### Modified Delphi method [11]

The modified Delphi method relies on group consensus to increase content validity in the setting of limited data. An iterative process, it combines an initial literature review with repeated rounds of voting by stakeholders to achieve group consensus. Incorporating a RAND (Research and Development)/UCLA (University of California- Los Angeles) Appropriateness Method (RAM) also includes face-to-face discussion, which is lacking from the traditional Delphi method. First, stakeholders identify the problem and perform a literature search. A questionnaire of best practices is developed and sent to experts in the field, who then anonymously score the best practices based on importance and relevance for inclusion into the desired guidelines. Experts are defined as 20–40 individuals from diverse geographic regions and with subject area knowledge [12]. Stakeholder responses are tallied after each round. The process is then repeated in a moderated, face-to-face meeting during which the panel discusses items that have not yet reached consensus. The RAM method also allows for modification of the technique in question to attempt to gain consensus during this discussion. Upon completion of discussion, panelists again rate the remaining items. Consensus is achieved if mean scores cross a certain threshold, which is determined by the researchers.

## Methods

We conducted a three-step modified Delphi process between November 2018–February 2019, consisting of a methodological assessment, a rating evaluation, and a face-to-face consensus meeting. We received approval from the Institutional Review Board for Baylor College of Medicine and Affiliated Hospitals (Protocol Number: H-43780). Given the study was minimal risk, informed consent was not required.

We contacted specialists in) the prehospital arena (paramedics, flight nursing, and EMS medical director), anesthesia, emergency medicine, obstetrics/gynecology, maternal-fetal-medicine, family medicine, neonatology, pulmonary critical care, nursing, certified nurse midwifery and cardiology. We invited subject matter experts in these fields as we felt these fields were most likely to come into contact with a patient in MCA. We invited them to join the panel based on their leadership positions on national committees/panels, or publications in related subject matter. Of the 25 experts invited; 22 individuals agreed to serve as panelists. Of those, almost half are women (42%), nearly half actively treat pregnant patients (46%), nearly a fifth represent those who provide out-of-hospital care (19%), and more than half (54%) represent in-hospital sectors.

We first established a baseline of the current evidence-based techniques used to resuscitate pregnant patients in cardiac arrest. Using a web-based assessment tool, 10 of the 12 invited experts and four of the clinician-investigators assessed the methodological quality of the AHA statement (AGREE II; 2010–2014, Hamilton, ON, Canada). Using a seven-point Likert scale, they assessed six quality domains of the AHA statement: scope and purpose; stakeholder involvement; rigor of development; clarity of presentation; applicability; and editorial independence (scale rankings one = strongly disagree to seven = strongly agree).

After reviewing the literature, internal investigator assessments, and expert assessment of the AHA statement, we developed and assigned a class of recommendation and level of evidence [13] to 12 pregnancy-specific resuscitation techniques for expert consideration. We categorized these into four themes: (1) resuscitative cesarean delivery, (2) point-of-care ultrasound, (3) extracorporeal cardiopulmonary resuscitation, and (4) targeted temperature management. We then performed two modified Delphi rounds to gain consensus on relevant pregnancy-specific technique statements.

### Round 1

First, the four investigators and 19 of the invited 22 experts used a seven-point Likert scale to rank the 12 technique statements through a secure, web-based data collection software (REDCap at Baylor College of Medicine) (Tables 2, 3 and 4). Investigators brought those that met the a priori consensus threshold to the face-to-face consensus meeting for facilitated discussion. An a priori determination of consensus to support the recommendation included an average score of 5.0 or greater; if any key question had a score of 1 “dangerous or inappropriate” it was automatically reviewed in round two. We calculated an average ranking score for each statement and incorporated reviewer comments for statement modifications.

**Table 2:** Likert scale to rank summary statements using modified RAM process

Ordinal Scale	Ranking	Description
0	No ranking	I do not have sufficient information or I am not an expert in this area and therefore cannot make a determination on the ranking.
1	Dangerous/Inappropriate	This recommendation is inappropriate and is actually dangerous to the patient or other health care providers. This recommendation should be removed from consideration for incorporation into the guideline.
2	Not important/Remove from consideration	This recommendation is inconsequential or of so little importance that it should be removed from consideration for incorporation into the guideline.
3	Less important	This recommendation is the lowest priority recommendation. It should only be considered for incorporation into the guideline with further discussion and consensus.
4	Average importance	This recommendation is moderately important. It should only be considered for incorporation into the guideline with further discussion and consensus.
5	More important	This recommendation is important and should be considered for incorporation into the guideline.
6	Extremely important	This is a critical, life-saving recommendation. Without incorporation of this recommendation into the current guidelines, the life of the mother may be lost.

**Table 3:** First round summary statements reviewed by expert panel

1. Training emergency department physicians in perimortem cesarean delivery (PMCD) is recommended so that PMCD can be immediately performed upon arrival to the hospital for out-of-hospital maternal cardiac arrest without return of spontaneous circulation (ROSC) (Class I; Level of Evidence C).	
2. PMCD should be immediately performed in a pregnant patient with a fundus height at or above the umbilicus with a non-shockable rhythm (versus proceeding with standard ACLS then PMCD after 4 minutes as would be recommended in pregnant patients with a shockable rhythm) (Class I; Level of Evidence C).	
3. The term “perimortem cesarean delivery” should be replaced with the term “resuscitative hysterotomy” to more correctly describe the purpose/indication and increase the sense of urgency for performing this procedure.	
4. First responders should initiate and maintain bag-mask-valve (BMV) techniques until arrival at a hospital with a more experienced laryngoscopist.	
5. Emergency Medical Services (EMS) should deploy highly specialized paramedics in addition to regular EMS crew in cases of suspected maternal cardiac arrest.	
6. The use of a ketamine-based anesthesia package should be considered for patients with ROSC who have undergone PMCD in settings without immediate anesthesia availability (Class IIb, Level of Evidence C).	
7. The use of extracorporeal life support (ELS, or eCPR) should be strongly considered for management of maternal cardiac arrest complicated by refractory cardiopulmonary resuscitation (CPR) in an extracorporeal membrane oxygenation (ECMO) center with capacity to care for critically ill pregnant patients (Class IIa; Level of Evidence C).	
8. The use of ELS or eCPR should be considered for organ procurement in pregnant patients post-arrest with circulatory determination of death (Class llb; Level of Evidence C).	
9. Where available, point-of-care ultrasound (POC-US) should be used in the management of maternal cardiac arrest for identification of an intrauterine pregnancy and quick determination of gestational age to guide decision making on PMCD (Class IIa; Level of Evidence C).	
10. POC-US should be considered for use during maternal cardiac arrest in emergency protocols for identification of potentially reversible causes of cardiac arrest, identification of cardiac contractility activity without palpable pulse for clinical reclassification of pulseless electrical activity, and identification of the absence of cardiac contractility where further attempts at resuscitation may be unsuccessful* (Class IIa; Level of Evidence C). *POC-US should not interfere with CPR, thus should only be performed during brief pauses in CPR.	
11. The use of POC-US by prehospital providers for diagnosis and management of maternal cardiac arrest should only be utilized in research protocols (Class IIa, Level of Evidence C).	
12. We recommend AGAINST the routine prehospital cooling of pregnant patients after ROSC with rapid infusion of cold intravenous fluids (Class III: No Benefit; Level of Evidence A).

**Table 4:** OBLS workgroup scores for each domain of the agree II assessment and overall approval of the 2015 AHA statement of managing cardiac arrest in pregnancy

Domain	Focus	Score
Domain 1: Scope and Purpose	Evaluates the overall aim, specific health question, and target population of the guideline.	82%
Domain 2: Stakeholder Involvement	Evaluates the degree to which the appropriate stakeholders developed the guidelines and represents the views and preferences of the target population	58%
Domain 3: Rigor of Development	Relates to the process used to gather and synthesize evidence including grading and summarizing, as well as the methods to formulate recommendations and to update them	72%
Domain 4: Clarity Of Presentation	Evaluates the presentation and format of the guidelines including language, structure, and format	90%
Domain 5: Applicability	Evaluates the consideration of likely facilitators or barriers to implementation, strategies to address them, and resources needed to apply the guidelines	65%
Domain 6: Editorial Independence	Is concerned with the guidelines being formulated without competing interests	85%
Overall Assessment	Rating of the overall quality of the guideline and whether the guideline would be recommended for use in practice	75%
Approve	Without modifications	57%
	With modifications	43%
	Total	100%

### Round 2

We held the face-to-face meeting on January 31–February 1, 2019. All 22 of the invited experts attended. We presented the individual statements and a summary of supporting evidence and facilitated a discussion to solicit perspectives. Our flexible agenda allowed for discussion and questions that followed. When differences resulted from the specific wording of the pregnancy-specific technique statements, we discussed, revised, and agreed on the rewording. Participants discussed all statements again, giving experts a chance to reconsider their earlier rating considering their colleagues’ views. We made all revisions and then provided participants with an online anonymous audience response system (Poll Everywhere, San Francisco, CA). Experts ranked the statements using a six-point Likert scale and answered, “Should this statement be incorporated into a future curriculum?” To be considered for affirmation and inclusion into the curriculum, an a priori result of 4.0 or greater, and > 80% consensus was set. A pre and post T-test was performed for each statement to determine the effect of face-to-face consensus discussion on change in ranking.

### Affirmation

Techniques that met preset criteria advanced to final affirmation. Via REDCap, participants individually and anonymously voted to include the pregnancy-specific resuscitation technique in the final overall curriculum priorities.

## Results

### Assessment of the AHA statement

Rating the acceptability, usability, and feasibility of the AHA statement, experts scored stakeholder involvement level low (58%) and clarity of presentation high (90%). They ranked the other four domains (scope and purpose, rigor of development, applicability, and editorial independence) greater than 72%. Reviewer comments for suggested modifications were collected and summarized into several common themes listed in Table 5.

**Table 5:** Expert reviewer-suggested modifications to the 2015 AHA statement

Could have definitely benefited from more stakeholders among patients, organizations	
There are out of hospital considerations which are not clearly addressed for maternal cardiac arrest	
Due to the lack of clear evidence this statement represents the most current knowledge and expert consensus to manage maternal cardiac arrest	
Outdated areas include the use of vasopressin and information related to post arrest hypothermia	
The section on EMS care should be further developed	
More specific direction on perimortem cesarean delivery (PMCD) should be included (including potential operators)	

### Modified RAM Delphi method

After the first round of consensus, only one statement met the predefined rank (> 5). While the remainder of the statements had a “high” (> 4) or “moderate” (> 3) level of consensus, they did not meet the threshold rank (Table 6).

**Table 6:** Rankings and Standard Deviations for Statements before and after Expert Panel Meeting using the modified RAM process (pre = ranking + SD after first round, prior to Expert Panel meeting, post = ranking +SD after second face-to-face consensus round at Expert Panel Meeting)

Statements	Pre Mean	Post Mean	Pre SD	Post SD
Training emergency department physicians in perimortem cesarean delivery (PMCD) is recommended so that PMCD can be immediately performed upon arrival to the hospital for out-of-hospital maternal cardiac arrest without return of spontaneous circulation (ROSC).	5.17	5.74	1.03	0.45
PMCD should be immediately performed in a pregnant patient with a fundus height at or above the umbilicus with a non-shockable rhythm (versus proceeding with standard ACLS then PMCD after 4 minutes as would be recommended in pregnant patients with a shockable rhythm).	4.78	5.35	1.13	0.80
The term “perimortem cesarean delivery” should be replaced with the term “resuscitative hysterotomy” to more correctly describe the purpose/indication and increase the sense of urgency for performing this procedure.	4.87	5.18	0.97	1.39
First responders should initiate and maintain bag-mask-valve (BMV) techniques until arrival at a hospital with a more experienced laryngoscopist arrives	4.57	1.92	1.59	1.29
EMS should deploy highly specialized paramedics in addition to regular EMS crew in cases of suspected maternal cardiac arrest.	4.87	2.52	1.32	0.98
The use of a ketamine-based anesthesia package should be considered for patients with return of spontaneous circulation (ROSC) who have undergone PMCD in settings without immediate anesthesia availability.	4.22	3.56	1.17	1.45
The use of extracorporeal life support (ELS, or eCPR) should be strongly considered for management of maternal cardiac arrest complicated by refractory cardiopulmonary resuscitation (CPR) in an extracorporeal membrane oxygenation (ECMO) center with capacity to care for critically ill pregnant patients.	4.86	5.40	0.94	0.76
The use of ELS or eCPR should be considered for organ procurement in pregnant patients post-arrest with circulatory determination of death.	4.33	4.53	0.97	0.77
Where available, point-of-care ultrasound (POC-US) should be used in the management of maternal cardiac arrest for identification of an intrauterine pregnancy and quick determination of gestational age to guide decision making on PMCD.	4.26	4.96	1.29	0.75
POC-US should be considered for use during maternal cardiac arrest in emergency protocols for identification of potentially reversible causes of cardiac arrest, identification of cardiac contractility activity without palpable pulse for clinical reclassification of pulseless electrical activity, and identification of the absence of cardiac contractility where further attempts at resuscitation may be unsuccessful.	4.90	4.78	0.89	0.85
The use of POC-US by prehospital providers for diagnosis and management of maternal cardiac arrest should only be utilized in research protocols.	3.87	4.30	1.46	1.02

Following the second round, nine of the 11 techniques met the predetermined rank for affirmation (> 4). Following participant discussion, experts scored three of these lower than they had in the first round. Investigators removed these methods from further consideration.

## Discussion

We obtained consensus among a diverse stakeholder group of experts regarding the most current evidence-based techniques to treat maternal cardiac arrest successfully. Experts agreed on themes related to resuscitative cesarean delivery, point-of-care ultrasound, extracorporeal cardiopulmonary resuscitation, and targeted temperature management. We hope that clinicians will incorporate these critical techniques to treat maternal cardiac arrest into future simulation-based education. (Table 7).

**Table 7:** Third round final statements affirmed for OBLS curriculum

1. Use ‘resuscitative cesarean delivery’ (RCD) instead of ‘perimortem cesarean delivery.’	
2. Providers staffing emergency departments should be trained in resuscitative cesarean delivery (RCD).	
3. Perform resuscitative cesarean delivery (RCD) immediately in a pregnant patient with a fundus height at or above the umbilicus with a non-shockable rhythm.	
4. The use of extracorporeal membrane oxygenation (ECMO, or eCPR) may be considered for the management of maternal cardiac arrest when there is no return of spontaneous circulation (ROSC).	
5. The use of extracorporeal life support (ELS or eCPR) should be considered for organ procurement in pregnant patients post-arrest after circulatory determination of death.	
6. Where available and when pregnancy stage and gestational age is uncertain, point of care ultrasound (POC-US) may be used in the management of maternal cardiac arrest for identification of an intrauterine pregnancy and quick determination of gestational age to guide decision making on resuscitative cesarean delivery (RCD).	
7. In maternal cardiac arrest with return of spontaneous circulation (ROSC), consider using point of care ultrasound (POC-US) in emergency protocols for identification of potentially reversible causes of cardiac arrest.	
8. Where proper training and resources are available, prehospital providers may use point of care ultrasound (POC-US) for diagnosis and management of maternal cardiac arrest.	

The study’s strengths include a robust literature search and rigorous process to generate an initial set of relevant, pregnancy-specific resuscitation techniques. Additionally, this systematic and thorough process brought together a multidisciplinary panel of experts representing out-of- and in-hospital settings in all North America regions. We believe this is the most diverse group of experts that has gathered around this specific topic area.

Limitations include our directing experts to select “0” if they did not feel qualified to rank a statement. They may have answered a question instead of selecting “0”, introducing bias.

Our decision to use anonymous voting was to limit bias. However, because we chose not to link answers to specific individuals, we were unable to calculate analytic statistics of individual member responses during the consensus rounds. Although we chose experts to represent diverse expertise and backgrounds, they may not adequately represent the full spectrum of views.

One other possible limitation is the rare nature of the topic of MCA. While best practice statements were based on a robust literature search it was primarily low-level evidence papers, which included multiple case reports and studies.. Future metrics to determine the validity of the best practices will also most likely rely heavily on simulation data due to the overall low volume of MCA. Low volume aside, there is still great benefit to assembling these best practices, as research has also shown significantly improved maternal response to resuscitation in MCA, particularly when they are applied promptly and aggressively.

## Conclusions

The results of this study provide the foundation to develop an optimal, long-term strategy to treat cardiac arrest in pregnancy through simulation-based training. We propose these nine priorities for standard practice, curricula, and guidelines to treat maternal cardiac arrest and hope they serve as a foundation for a future educational curriculum.

### Supplement

#### Resuscitative cesarean delivery (formerly Perimortem cesarean delivery)

1. Use the term ‘resuscitative cesarean (or vaginal) delivery’ (RCD) instead of ‘perimortem cesarean delivery’ to more correctly describe the purpose/indication and increase the timeliness and sense of urgency for performing this procedure (Class I; Level of Evidence C).
  Perimortem cesarean delivery was originally used to describe the last-ditch effort to save the fetus in a dying pregnant patient. However, we now understand that the technique to rapidly evacuate uterine contents may actually improve maternal resuscitation efforts by increasing cardiac output through the reduction of aortocaval compression and the resultant autotransfusion from the decompressed uterus [14–16]. As it incorporates delivery of the fetus and placenta, “resuscitative cesarean delivery” (RCD) is a more accurate description of the procedure, making it instantly recognizable to non-obstetric staff who may be assisting in the resuscitation. It reflects the mutual optimization of resuscitation efforts that would potentially provide earlier and more substantial benefit to both the pregnant woman and fetus.
2. Perform an RCD immediately in a pregnant patient with a fundal height at or above the umbilicus with a non-shockable rhythm (Class IIb; Level of Evidence C).
  Delivery of the fetus early in a maternal cardiac arrest following the absence of ROSC results in a high probability of ROSC in the pregnant patient, more than 50% in some studies, by improving maternal cardiac output through the reduction of aortocaval compression, and the resultant autotransfusion from the decompressed uterus [14, 15]. A recent study of 462 women with maternal cardiac arrest found that only 11.2% presented with a shockable rhythm [16, 17]. This highlights the need for simultaneous RCD preparations with ongoing cardiopulmonary resuscitation (CPR) efforts, and immediate performance of an RCD if the maternal cardiac rhythm is non-shockable and the uterus is at or above the umbilicus, regardless of gestational age or the presence of fetal cardiac activity.
3. Train providers staffing EDs in RCD (Class IIb; Level of Evidence C)
  Inherent in our recommendations is the recognition that medical professionals staffing emergency departments and hospitals who do not provide obstetric care need to have the capability to perform a resuscitative cesarean delivery to improve maternal survival within the 5 min window from arrest (if in-hospital) or at the time of admission to the Emergency Department if the maternal cardiac arrest occurred out-of-hospital. It is imperative that physicians in all fields that staff emergency departments learn the proper techniques of resuscitative cesarean delivery through proctored clinical experience and maintain clinical competency via simulation [18–20].

### Extracorporeal cardiopulmonary resuscitation

#### Recommendations

1. Consider ECPR to manage maternal cardiac arrest when there is no ROSC after RCD or for refractory CPR where the uterus has not yet reached the umbilicus and the patient is in an extracorporeal membrane oxygenation center with the capacity to care for critically ill pregnant patients (Class IIa; Level of Evidence C).
  Extracorporeal cardiopulmonary resuscitation (ECPR) has emerged as a beneficial intervention when sustained ROSC is not achieved. ECPR maintains organ perfusion while the underlying etiology of the cardiac arrest is determined and treated. As of 2018, we identified 36 cases of ECPR use in maternal cardiac arrest, with many cases resulting in maternal survival [18, 19, 18–23]. ECPR has been used for a variety of etiologies in maternal cardiac arrest including massive pulmonary embolism, severe cardiomyopathy/cardiac disease, amniotic fluid embolism, sepsis, and postpartum hemorrhage in which CPR failed to result in sustained ROSC [19, 24–28]. Although ECPR has been applied at a variety of gestational ages, it is generally applied after RCD in the second half of pregnancy [24]. When ECPR has been applied in the setting of maternal cardiac arrest without ROSC, survival rates to discharge have been reported as high as 77% of women and 67% of fetuses [21]. These rates compare very favorably to a recently published overall maternal survival rate to discharge of 40.7% after cardiac arrest [17].
2. Consider ECPR for organ procurement in pregnant patients, post-arrest with circulatory determination of death (Class IIb; Level of Evidence C).
  If a pregnant woman is unsuccessfully resuscitated, ECPR has been shown to be effective for the procurement of organs in the circulatory determination of death [29, 30]. Reproductive-age women who have maternal cardiac arrest with unsuccessful CPR are generally younger and in better health, making them ideal candidates for organ procurement. Moreover, the results of some studies have highlighted the favorable impact of organ donation on the reduction of sorrow from the loss of a loved one, and the concept that “live organs in recipients’ body somehow reflect immortality.” [31, 32].
3. Report all pregnant women who receive ECPR in the setting of maternal cardiac arrest, regardless of outcome, in the Extracorporeal Life Support Organization (ELSO) registry (https://www.elso.org/Registry.aspx) (Class IIb, Level of Evidence C).
  Future directions on the application of ECPR for refractory CPR in pregnant patients and the refinements to the use of ECPR in this population will depend on registry reporting and continued re-evaluation of observational trials and case reports or series.

### Point of care ultrasound

#### Recommendations

1. Where available, POC-US should be used in the management of maternal cardiac arrest for identification of an intrauterine pregnancy and quick determination of gestational age to guide decision making on RCD (Class IIa; Level of Evidence C)
  Currently, focused assessment of sonography for trauma (FAST) protocols and extended FAST (eFAST) protocols used in the setting of trauma do not incorporate an examination of the abdominopelvic area to detect pregnancy in reproductive-age women. However, at least one case demonstrated a timely diagnosis of intrauterine pregnancy during a FAST exam in a reproductive-age female with gunshot wounds to the chest [33]. Adding POC-US for the rapid identification of pregnancy to FAST and eFAST protocols for this patient population may decrease time to RCD, which may optimize outcomes.
  There are also certain circumstances where the estimation of gestational age using fundal height alone is inadequate, such as extreme obesity. In this setting it may be necessary to use POC-US to determine gestational age quickly. Emerging data shows that providers with limited ultrasound training can effectively perform swift and targeted POC-US to determine gestational age [34, 35]. A single feasibility study evaluated the ability to teach emergency medicine providers (residents, fellows, sonographers) limited goal-directed obstetrical ultrasound to estimate gestational age. The learners quickly and accurately estimated gestational age in second and third trimester pregnancies using ultrasound measurement of biparietal diameter (BPD) [35]. Recently, MacCarthur et al. recommended incorporating a fetal FAST exam in trauma protocols in pregnant women and using femur length to determine gestational age quickly [36, 37]. Using POC-US to estimate gestational age, when indicated, appears promising.
2. Consider POC-US during maternal cardiac arrest in emergency protocols for identification of potentially reversible causes of cardiac arrest, identification of cardiac contractility activity without palpable pulse for clinical reclassification of pulseless electrical activity, and identification of the absence of cardiac contractility where further attempts at resuscitation may be unsuccessful (Class IIa; Level of Evidence C).
  Point-of-care ultrasound (POC-US) is currently used across emergency protocols to identify potentially reversible causes of cardiac arrest, cardiac contractility activity without a palpable pulse for clinical reclassification of pulseless electrical activity, and the absence of cardiac contractility where further tries at resuscitation may be unsuccessful. Despite the differences in maternal physiology from non-pregnant adults, many case reports highlight the successful application of POC-US to manage maternal cardiac arrest [34, 38–40]. POC-US has been used for earlier diagnosis and treatment of pulmonary embolism, pneumothorax, and pericardial effusion in out-of-hospital and in-hospital maternal cardiac arrest.
  Where properly trained and resources are available, out-of-hospital practitioners may use POC-US for diagnosis and management of maternal cardiac arrest (Class IIa; Level of Evidence C).
  POC-US has also been used for the rapid identification of an intrauterine pregnancy to help guide resuscitation management [34]. The knowledge compels EMS providers to transport the patient to the most appropriate care location and mobilize advanced resources to perform an RCD. This more rapid care flow and timing may improve maternal outcomes.

### Targeted temperature management

#### Recommendations

1. Current AHA recommendation: TTM should be considered in pregnancy on an individual basis (Class IIb; Level of Evidence C).
  The routine use of targeted temperature management (TTM) in cardiac arrest is supported by the AHA based on the positive results of two large randomized trials in non-pregnant adults [41–43]. As there were only two case reports of TTM use in maternal cardiac arrest at the time of publication [44, 45] and lingering concerns for bleeding because of impaired systemic coagulation, the 2015 AHA statement recommended using post-arrest cooling on an individualized basis, and, if used, follow the same current protocol as for non-pregnant) [3]. Recently, several case reports noted using post-arrest TTM in the setting of maternal cardiac arrest with favorable maternal outcomes [5, 46–51]. This mounting evidence bolsters the current AHA position supporting the use of post-arrest TTM on an individual basis in pregnancy, with particular focus on the potential for bleeding complications during TTM.
2. We recommend against the routine use of out-of-hospital induction of cooling after ROSC in pregnancy (Class III: No Benefit, Level of Evidence A)
  The 2015 AHA Executive Summary for Cardiopulmonary Resuscitation recommends against the use of out-of-hospital cooling in cardiac arrest in non-pregnant adults [41]. A recent large meta-analysis of seven randomized controlled trials demonstrated potential harm from out-of-hospital induction of cooling. Out-of-hospital induction of cooling may increase the risk of cardiac re-arrest. We support this AHA recommendation in the setting of pregnancy as well.

## Data Availability

All data generated or analyzed during this study are included in this published article.

## Abbreviations

MCA: Maternal cardiac arrest
AHA: American Heart Association
RAM: RAND/UCLA Appropriateness Method
ACLS: Advanced cardiac life support
PMCD: Perimortem cesarean delivery
ROSC: Return of spontaneous circulation
ECPR: Extracorporeal cardiopulmonary resuscitation
POC-US: Point-of-care ultrasound
RCD: Resuscitative cesarean delivery
ECMO: Extracorporeal membrane oxygenation
BMV: Bag-mask-valve
